# Real-Space Pseudopotential Method for the Calculation of 1*s* Core-Level Binding Energies

**DOI:** 10.1021/acs.jctc.2c00474

**Published:** 2022-08-29

**Authors:** Qiang Xu, David Prendergast, Jin Qian

**Affiliations:** † Chemical Science Division, Lawrence Berkeley National Laboratory, Berkeley, California94720, United States; ‡ Molecular Foundry, Lawrence Berkeley National Laboratory, Berkeley, California94720, United States

## Abstract

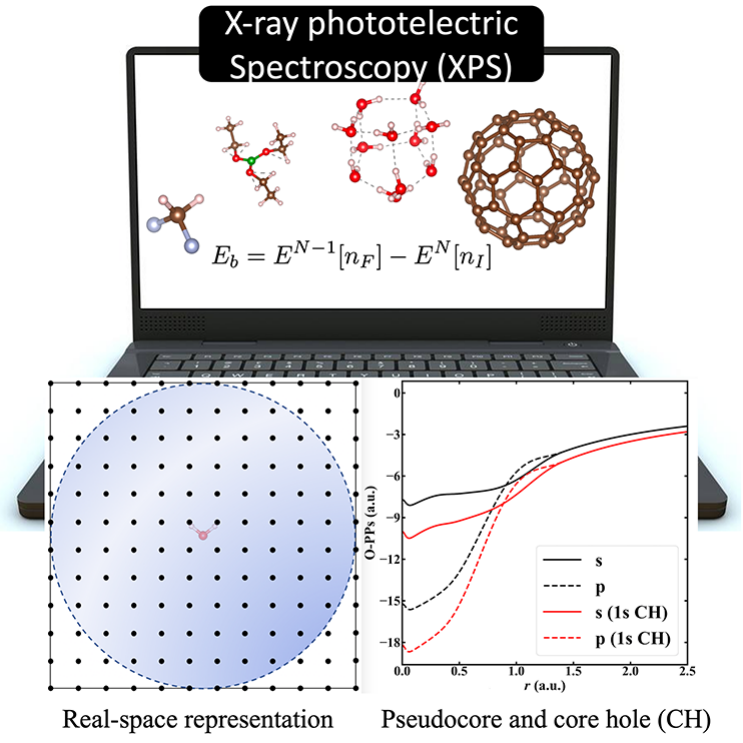

We systematically studied a real-space pesudopotential method for the calculation of 1*s* core–electron binding energies of second-row elements B, C, N, and O within the framework of Kohn–Sham density functional theory (KS-DFT). With Dirichlet boundary conditions, pseudopotential calculations can provide accurate core–electron binding energies for molecular systems, when compared with the results from all-electron calculations and experiments. Furthermore, we report that with one simple additional nonself-consistent calculation as a refinement step using a hybrid exchange-correlation functional, we can generally improve the accuracy of binding energy shifts, promising a strategy for improving accuracy at a much lower computational cost. The specializations in the present approach, combined with our efficient real-space KS-DFT implementation, provide key advantages for calculating accurate core–electron binding energies of large-scale systems.

## 1 Introduction

X-ray photoelectron spectroscopy (XPS) is a powerful characterization technique widely adopted in the context of physics, chemistry, and materials science.^1−8^ XPS chemical analysis relies purely on the measurement of core–electron binding energy (CEBE). The same element in different chemical environments can display quite distinct CEBEs. These chemically sensitive relative CEBEs are often referred to as binding energy shifts or chemical shifts.^9^ Binding energy shifts are keys for distinguishing the local structure or chemical environment around a given atom.^10,11^ Therefore, accurate estimates of CEBEs and chemical shifts are of great importance for analyzing and predicting the local elemental composition and structure in materials.

The CEBE (*E*_*b*_) can be obtained from *ab initio* calculations.^10,12,13^ The absolute CEBE is defined as the energy difference between the initial ground state and final core-hole state of the system^10^
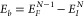
1where *E*_*F*_^*N*–1^ and *E*_*I*_^*N*^ are the exact total energies of the final core-hole state with (*N* – 1) electrons and initial ground state with *N* electrons, respectively. Unfortunately, it is not easy to obtain the exact total energies for intrinsically many-body systems. Alternatively, approximate theoretical methods are available to obtain the binding energies, such as 1) the one-particle eigenvalue evaluations from Koopman’s approximation^10,14,15^ or restricted open-shell Kohn–Sham using orbitals from a mixed energy estimate;^16^ 2) the Δ self-consistent field method (ΔSCF) based on Hartree–Fock theory^17−28^ or Kohn–Sham density functional theory (KS-DFT);^29−32^ 3) post-HF methods, such as configuration interaction,^33,34^ coupled cluster method,^35,36^ and equation of motion coupled cluster method;^37^ and 4) the *GW* approximation based quasiparticle methods.^38−41^

In this manuscript, we present a clear theoretical strategy for binding energy calculations within the ΔSCF scheme of real-space KS-DFT. The core-excited final states are described using a suite of specially tuned core-hole pseudopotentials (PPs), in parallel with our recent successful development of Slater’s rule motivated Gaussian-type basis sets within the scheme of orbital-like-based KS-DFT.^13^ Pioneering works using the PP approach^29,42−53^ within periodic boundary conditions on sporadic systems demonstrated that the relative binding energy shifts (*ΔE*_*b*_ = *E*_*b*_ – *E*_*b*_^*ref*^, where *E*_*b*_^*ref*^ is the binding energy of an arbitrary reference system) from PP-based calculations are generally consistent with experiment, while the numerical convergence, transferability, generalizability, computational accuracy, and efficiency are critically worthy of further study.

## 2 Results and Discussion

### 2.1 Pseudopotential Binding Energy Estimates

The binding energy expression obtained by ΔSCF within KS-DFT can be written as
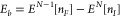
2where *E*^*N*–1^[*n*_*F*_] and *E*^*N*^[*n*_*I*_] are the total energy functionals of final and initial electron density, respectively. According to eq 2, the binding energy is calculated as the energy difference between two separate KS-DFT calculations. Note that the total energies should be obtained, in principle, from the KS-DFT calculations within an all-electron (AE) picture; however, the AE strategy becomes computationally intractable for large-scale calculations. In addition, special care must be taken in the AE final-state calculation to prevent variational collapse to the AE ground state. Alternatively, Birgersson et al.^48^ demonstrated that the relative binding energy shifts *ΔE*_*b*_ can be obtained from PP-based calculations. Here, we derive the absolute binding energies and their shifts from the cohesive energy *E*_*c*_
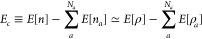
3where *N*_*a*_ is the number of atoms in the simulation systems. *n* and ρ denote the AE and PP electron densities, respectively, i.e., *E*[*n*_*a*_] and *E*[ρ_*a*_] are the AE and PP energies of the *a*-th isolated atom, respectively, which can be conveniently obtained from the PP generation step using the AE and PP solver.^54^ Thus, one may approximate the AE total energy from eq 3:
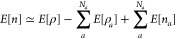
4

Combining eqs 2 and 4, one can obtain the binding energies from the PP-based calculations, because in principle, PPs can provide accurate cohesive energies. Furthermore, the PPs are able to reproduce the correct scattering behavior of the AE potentials,^54−57^ which guarantees the high transferability for the evaluation of the electron-pseudocore interaction energy in different chemical environments, even though the core electrons are excluded in PPs. Therefore, this special “error cancellation” provided by subtracting the core-excited atom from the core-excited full system energy leads to reasonable accuracy. Note that possible neglect of atomic open-shell characteristics tends to cancel out when calculating the relative binding energy shift (*ΔE*_*b*_), as shown in the Numerical Results section.

### 2.2 Computational Details

Two sets of benchmark data are provided for comparison: binding energies of B, C, N, and O elements from experiments,^9^ as well as binding energies from AE calculations using the Q-Chem package.^58^ Molecular structures and energies were optimized at the PBE level using Q-Chem^58^ with cc-pVTZ basis sets,^59^ whereas the ΔSCF approach within the maximum overlap method (MOM)^60^ was used for the calculations of AE binding energies. The calculations using Troullier–Martins PPs^61^ are performed using the real-space code ARES.^62^ For each CEBE calculation, two PPs with pseudocore and core hole are generated using the FHI98PP code^54^ for the initial and final state calculations, respectively. Under the fully screened core-hole assumption,^10^ only the traditional self-consistent iterations are required for both initial and final state calculations with no extra time costs.

PBE^63^ and B3LYP^64^ exchange-correlation functionals are used in our real-space KS-DFT calculations. Note that B3LYP implemented in ARES^62^ is only used as a refining step, as a nonself-consistent recalculation, using the PBE KS orbitals and electron density as inputs to obtain the total energy *E*[ρ] in eq 4 with the B3LYP hybrid functional.

### 2.3 Numerical Results

**Figure 1:**
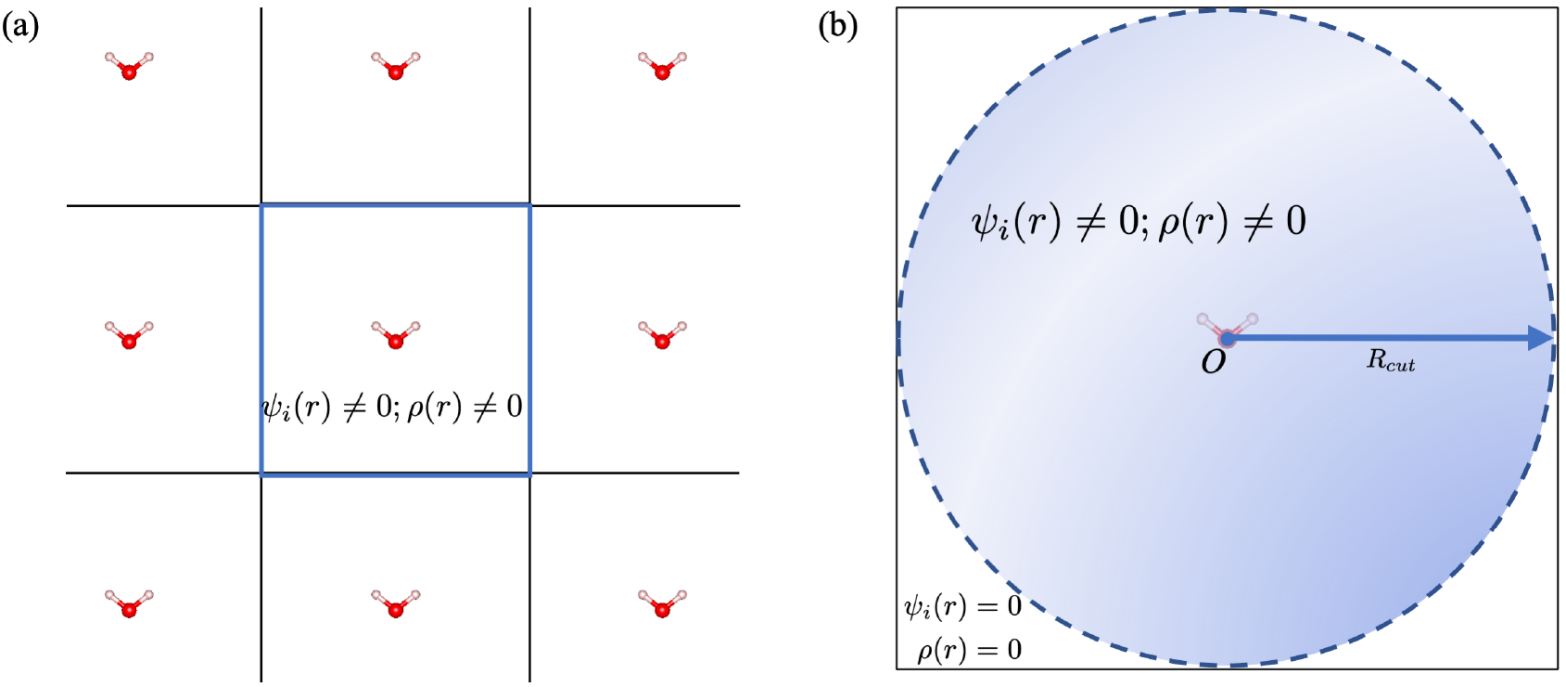
Schematic illustrations of the calculations under (a) PBC and (b) DBC.

The real-space implemented ARES package allows for easy selection of periodic boundary conditions (PBC) [Figure 1(a)] and Dirichlet boundary conditions (DBC) [Figure 1(b)]. To simulate the isolated molecule systems under PBC, a supercell padded with a large vacuum region is adopted to reduce spurious long-range electrostatic interactions between periodic images. However, it usually leads to difficulties converging the total energy with respect to the size of the supercell if there are strong electrostatic interactions between the periodic replicas, especially for charged systems, even if compensation charges (or background charges) were included in the unit cell. This is particularly relevant here, as the core-excited final states are charged, and, as such, this is one of the main disadvantages of calculating binding energies under PBC. Therefore, in practice, applications of plane-wave based packages for the calculations of CEBE must proceed with caution. The user must estimate the final state convergence with respect to the supercell size or adopt one of many correction schemes that mathematically counteract the slow long-range convergence of the Coulomb interaction.^65−68^ While for DBC, a reliable radius, *R*_*cut*_, of a spherical region is used to truncate the tails of the wave functions ({ψ_*i*_}) and electron density (ρ), whose values are zero beyond the spherical region. The values of the electrostatic potential at the boundary are calculated by the multipole expansion method,^69^ and the values in the simulated cell are calculated by solving the Poisson equation in real space.^69,70^ So far, the Kohn–Sham equations can be solved within the spherical region, making it feasible to model an isolated finite system. The definition of the neutral and ionized states is therefore precise in our calculation scheme.

**Figure 2:**
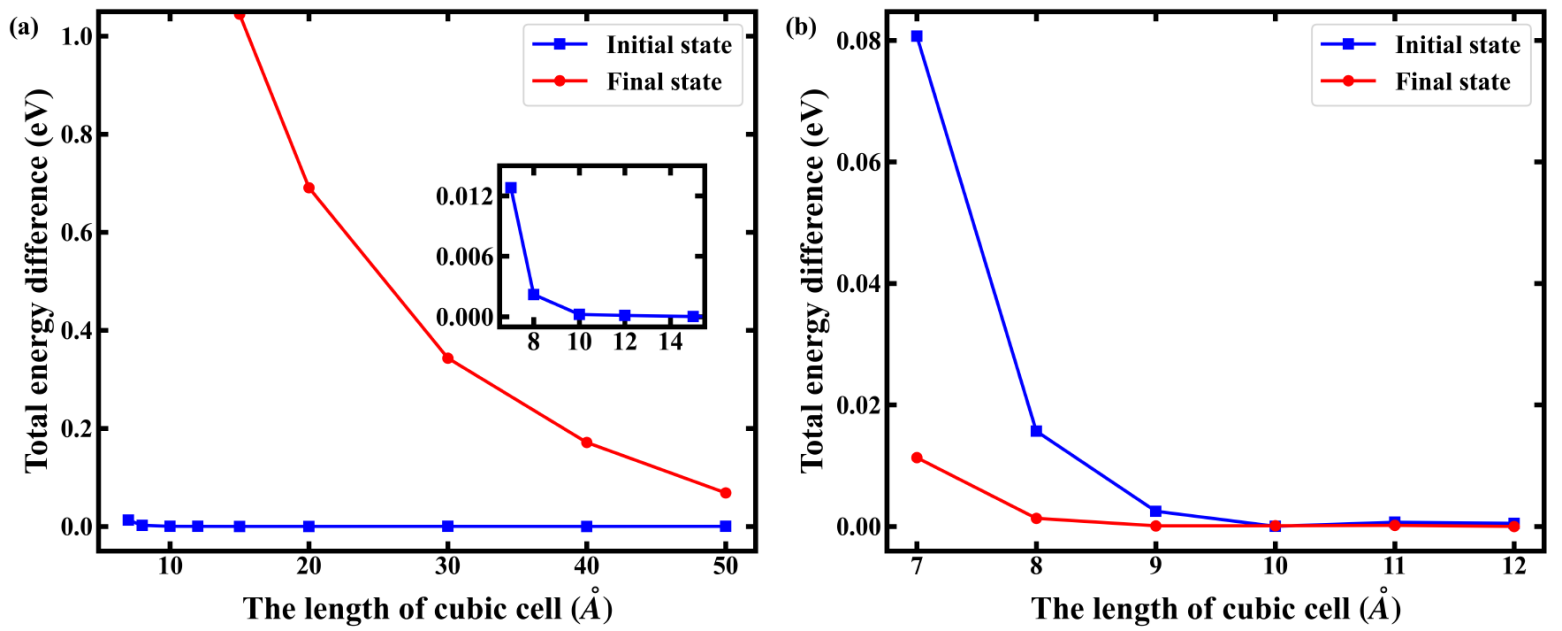
Total energy convergence with respect to the cell size of initial and final states for CH_4_ within (a) PBC and (b) DBC.

To explore the effect of boundary conditions on energy convergence, the initial- and final-state energy differences versus the length of the cubic cell for CH_4_ are calculated by KS-DFT using the PBE functional. These calculations were performed using the real-space code ARES^62^ under both PBC and DBC (Figure 2). As shown in Figure 2(a), it is difficult to converge using PBC for the charged final-state system even if more than 50 Å vacuum has been included, while the neutral initial-state calculation under PBC shows well converged results [the blue curve of Figure 2(a)]. Problems will arise in comparing the binding energy shifts of the selected element in the systems modeled using similar supercells that exhibit different dielectric constants. For the calculations under DBC, a cubic cell with a length of only 10 Å is required to arrive at a total energy convergence of less than 1 meV for both initial and final energies. Therefore, DBC is highly recommended in practice, and it is therefore adopted here for all the rest of the calculations.

Note that eq 4 is accurate in principle, but in practice, the effect of spin polarization for the isolated atom energies (E[ρ_a_] and E[n_a_]) is ignored in the standard PP generation step. For ground state PPs, the core orbitals typically define a spin-unpolarized system, and the PP is provided without spin dependence. In contrast, the core hole explicitly polarizes the core, which should separate the PP by spin channel, since it is nominally designed to reproduce the AE valence atomic energies. In fact, the absolute energy difference between spin-polarized and spin-unpolarized calculations of atoms with a core hole can be as large as several eV. As shown in Table 1, the AE-ion energy differences are ∼6–14 eV between spin-unpolarized and spin-polarized calculations. The findings are consistent with our expectation that the core-hole states are strongly spin-polarized. Fortunately, the error frequently only contributes to a constant shift for a given element, which is canceled out when computing relative binding energy differences (*ΔE_b_*). This implies that the effect is mostly limited to the core-excited atom. In addition, one can compensate for this constant shift by recalculating the atomic and ionic energies in the PP generation step to improve the absolute binding energy accuracy. As listed in Table 2, the atomic and ionic energies recalculated within spin polarization can generally improve the accuracy of absolute binding energies by 76%–89% with respect to the spin-unpolarized estimations (benchmarked by the results of AE-PBE). It can be expected that the spin-dependent PP^71^ based calculations may provide even more accurate results for the prediction of absolute binding energies in the future.

**Table 1:** Atomic and Ionic Energies (with 1*s*-Core Electron Missing) in eV Calculated by Using the FHI98PP Code within Spin Unpolarized and Polarized Schemes, Compared with the AE Solution as the Benchmark

element	spin	AE atom	AE-ion	PP atom	PP-ion
B	unpolarized	–669.486	–462.410	–70.483	–128.688
	polarized	–669.753	–469.348	–70.796	–129.236
C	unpolarized	–1027.632	–723.254	–145.943	–240.665
	polarized	–1028.869	–733.736	–147.314	–242.723
N	unpolarized	–1481.741	–1061.468	–262.446	–401.742
	polarized	–1484.864	–1075.962	–265.824	–406.408
O	unpolarized	–2040.902	–1486.074	–428.168	–619.405
	polarized	–2042.420	–1498.513	–429.765	–621.455

**Table 2:** Absolute Binding Energies (eV) Calculated by AE and PP Methods in Comparison with Experiments

cluster	1*s* core hole	exp.^9^	AE-PBE	PP-PBE	PP-PBE^a^
P(CH_3_)_3_BH_3_	B	192.93	192.26	198.04	191.60
CH_3_SiH_3_	C	290.31	289.78	296.71	288.16
(C_2_H_5_)_2_NH	N	404.58	403.84	411.94	401.86
S(CH_3_)_2_O	O	536.67	535.71	544.93	534.46

a The atomic and ionic energies recalculated within spin-polarized.

**Figure 3:**
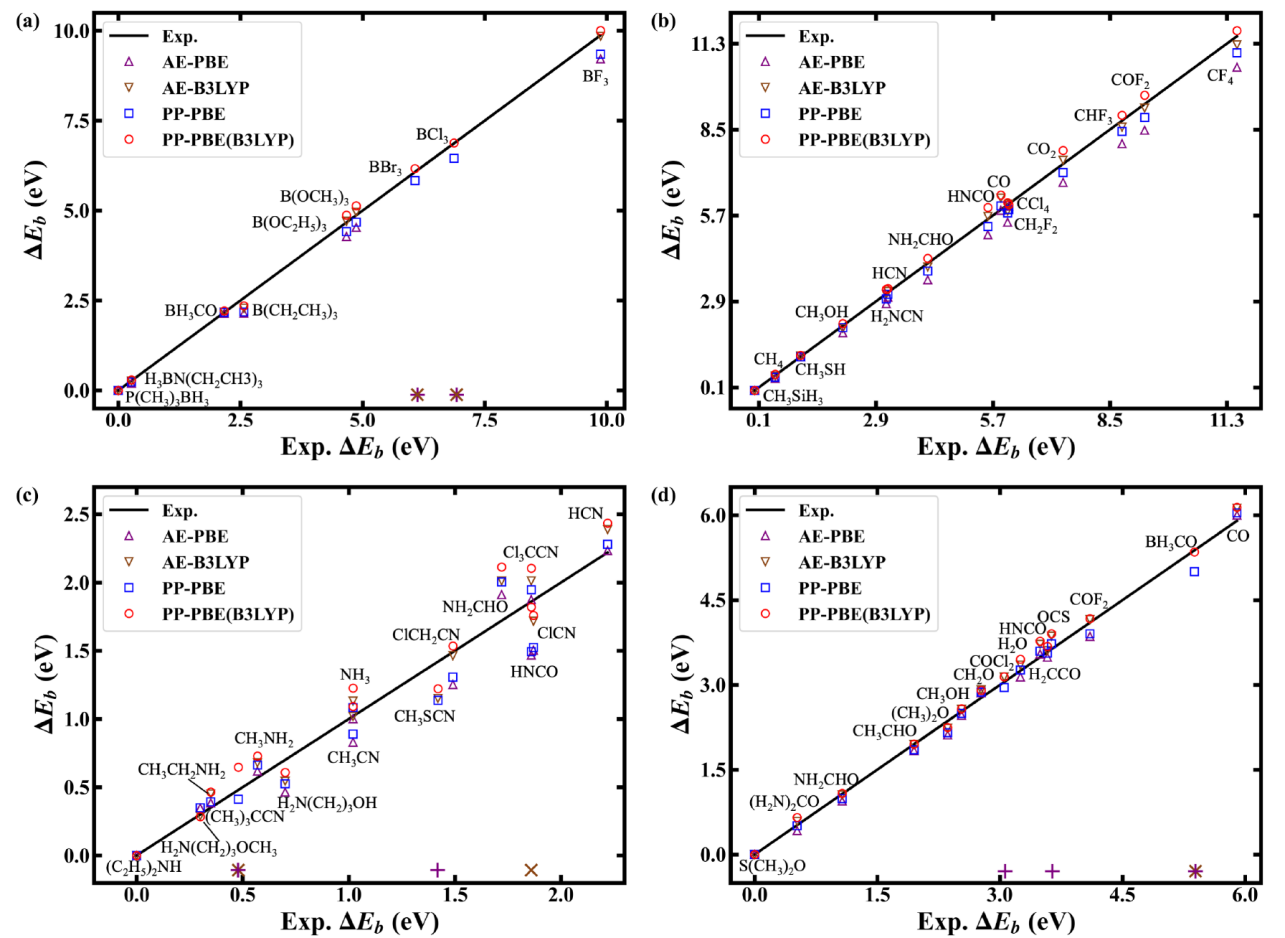
Binding energy shifts of molecules for (a) B, (b) C, (c) N, and (d) O core excitations, where + and × denote the problem of variational collapse for core-hole state calculations of AE-PBE and AE-B3LYP by MOM, respectively, or see Table S1 in the Supporting Information.

To access the accuracy of binding energy shifts predicted by KS-DFT within the PP scheme, the relative binding energies of a wide range of molecules with respect to P(CH_3_)_3_BH_3_, CH_3_SiH_3_, (C_2_H_5_)_2_NH, and S(CH_3_)_2_O for B, C, N, and O elements, respectively, are calculated by Q-Chem^58^ using MOM and by ARES^62^ under DBC. All values of *E*_*b*_ and *ΔE*_*b*_ can be found in Supplementary Table S1. The selection criteria of the presently chosen molecular test sets and the necessity of benchmark against molecules with diverse local charge were discussed in detail in ref (13) for validating the transferability and generalizability of CEBE method development. The calculated binding energy shifts in comparison with that of experiments are shown in Figure 3. All calculations by PP-PBE can reproduce the results by AE-PBE, which are generally able to give the same trends as the experiments. In addition, the non-SCF calculations by B3LYP after PP-PBE calculation, denoted as PP-PBE(B3LYP), yield similar results as those by AE-B3LYP. For the *ΔE*_*b*_ of N in Figure 3(c), the order of relative energies predicted by the PP method is admittedly deviating from the experiment, while consistent with those predicted by the AE approach. It is also worth noting that the PP-based scheme shows good numerical stability for all calculations, while the AE calculations within MOM sometimes face the problem of variational collapse for the final core-hole states.

In order to further quantify the accuracy of the binding energy shifts using the PP method, the mean absolute errors of *ΔE*_*b*_ are provided in Table 3. It is apparent that PP-PBE under real-space DBC has comparable accuracy as AE-PBE. Furthermore, the refined calculation scheme, using nonself-consistent B3LYP calculations (now implemented in ARES^62^) shows a consistent accuracy enhancement when compared to PP-PBE. Therefore, the additional refinement step by the hybrid functional is a useful and efficient approach to improve the predicted accuracy of binding energy shifts, see results in Table 3. In the Supporting Information, we provide detailed discussions about the refined binding energy shifts of N within the different coefficients of Hartree–Fock exchange in B3LYP (see Supporting Information Figure S1 and Table S2).

**Table 3:** Mean Absolute Error of the Molecules’ Binding Energy Shifts (eV) with Respect to the Experiments for the B, C, N, and O Elements

method	B	C	N	O
AE-PBE	0.27	0.38	0.14	0.11
AE-B3LYP	0.06	0.12	0.12	0.10
PP-PBE	0.23	0.20	0.15	0.11
PP-PBE(B3LYP)	0.11	0.18	0.14	0.12

**Figure 4:**
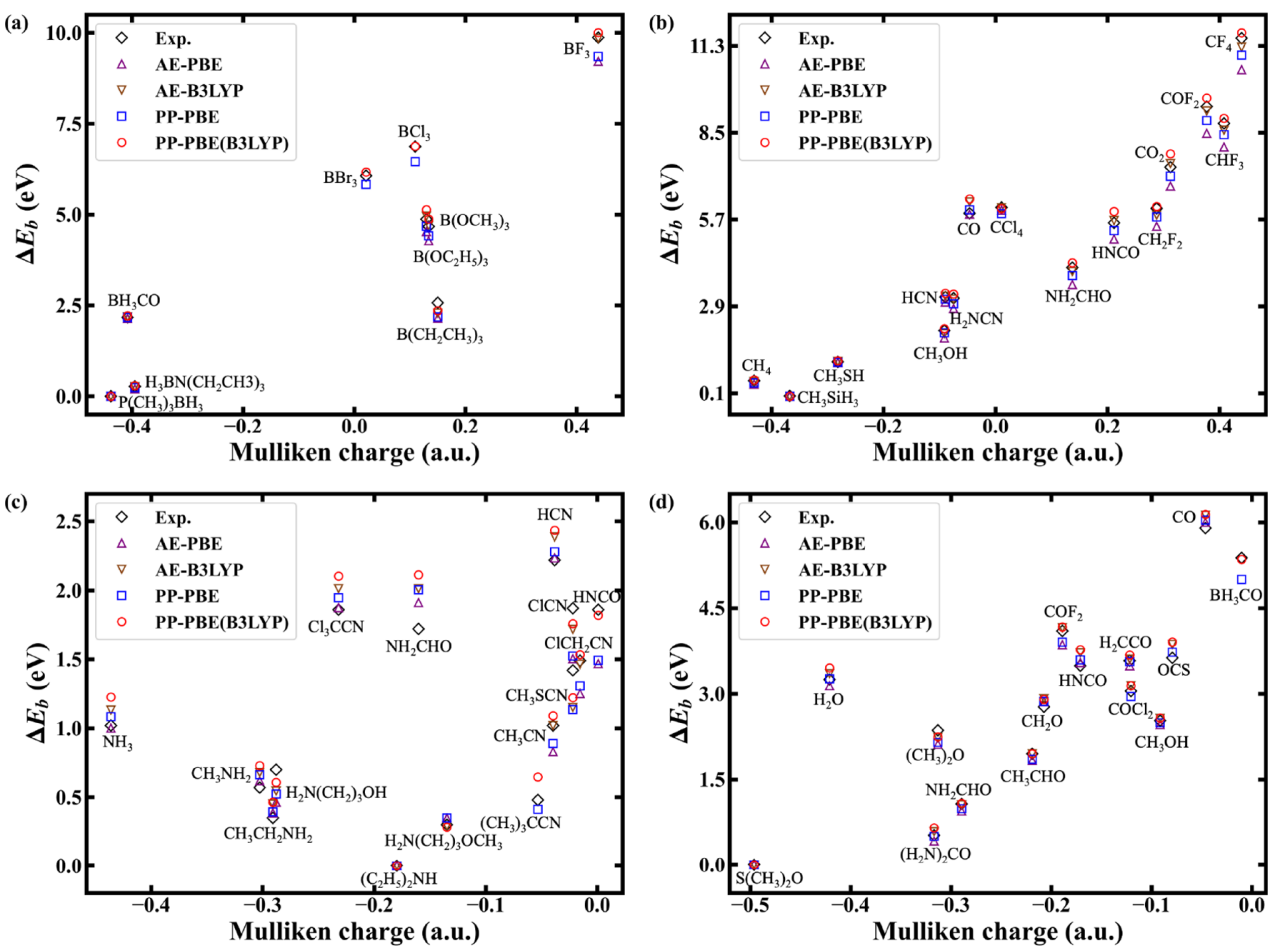
Binding energy shifts versus the Mulliken charges of molecules for (a) B, (b) C, (c) N, and (d) O core excitations.

We can rationalize the results by pointing out the relationship between local charges and binding energies.^13,72^ Core excitation for a more positively charged atom will generally result in a larger binding energy, for example, CF_4_ will have a binding energy 11.54 eV larger than CH_3_SiH_3_ (see Table S1 in the Supporting Information). Binding energy shifts versus the Mulliken charges on the B, C, N, and O elements are shown in Figure 4. The binding energy shifts and Mulliken charges are generally positively correlated. The weakly correlated relation between Mulliken charges and binding energy shifts for N-containing molecules in Figure 4(c) falls into a narrow energy range (within 2.5 eV) in the vertical axis. Similarly, a small energy range with weak correlations can also be observed in Figure 4(a), (b), and (d). Since the relationship between local charge and binding energy is not exactly linear,^13^ one could imagine that computational methods that work well for a narrow range of oxidation states might not be transferable to a broader context. Therefore, a comprehensive range of possible oxidation states for a given element is presently included to validate the transferability of our approach.

**Figure 5:**
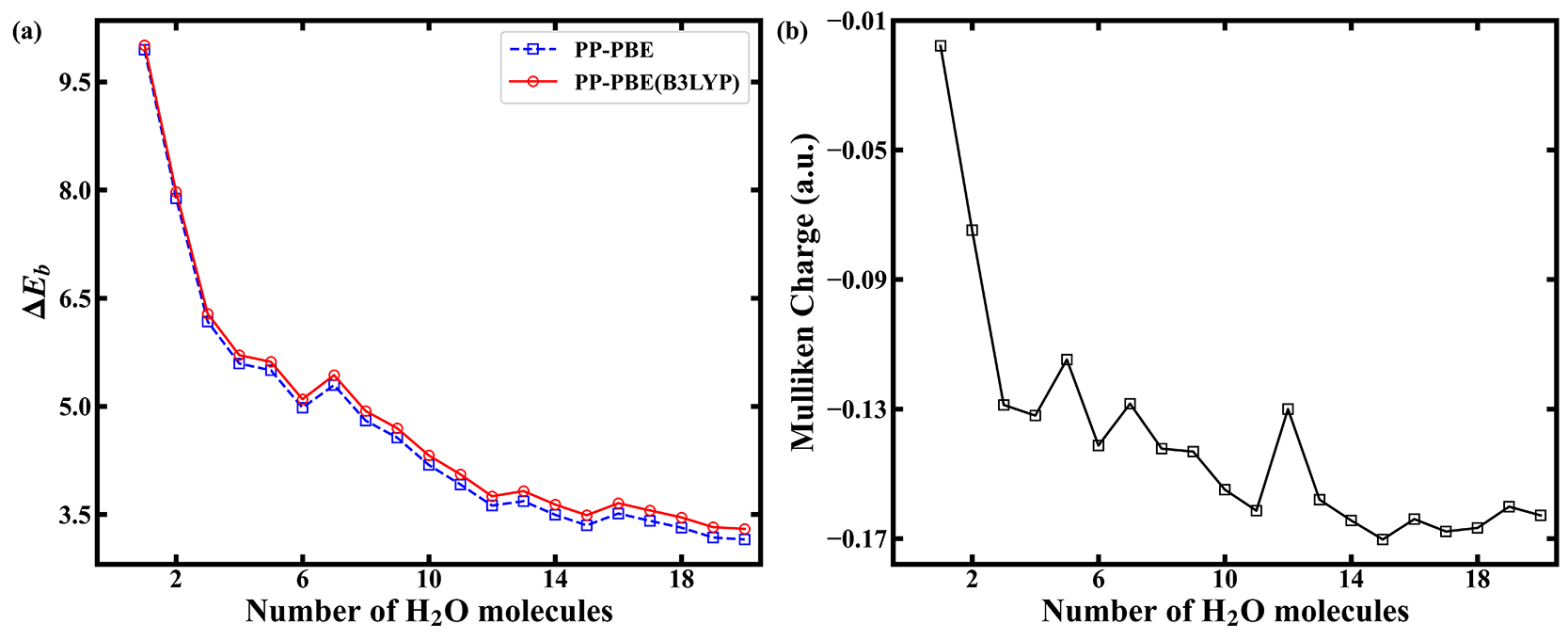
(a) The binding energy shifts of the O-1*s* core level for protonated water with respect to that of an isolated water molecule. (b) The Mulliken charge on the O atom in protonated water.

Lastly, we turn to a case study to demonstrate the capabilities of the current real-space KS-DFT PP approach. The nature of the proton in water is one of the most fundamental aspects of aqueous chemistry,^73−75^ dominating the behaviors in acid–base reactions. However, it is often difficult to characterize the local structure of the proton in an aqueous environment.^76^ XPS can be used as a surface sensitive technique to probe the local structure of the proton and its surrounding water molecules. We further calculated the O-1*s* binding energy shifts in protonated water with respect to a free water molecule [Figure 5(a)]. Global optimized structures [H_3_O^+^...(H_2_O)_*n*_, *n* ≤ 20] by anisotropic site potential^77^ were obtained from refs (78 and 79), and the O in protonated waters is highlighted in Figure S2 of the Supporting Information. Note that the initial state of a protonated water cluster is charged rather than neutral in its ground state. The results here show that the O-1*s* binding energy shifts in protonated water converge to about ∼3.5 eV as the number of H_2_O molecules increases to greater than 14 in the clusters considered. Again, we rationalize the results through the correlation with local charge. In Figure 5(b), the Mulliken charges also show a similar trend of convergence as the binding energy shifts. These results provide insight on atomic-scale screening events at the heart of aqueous chemistry, while providing theoretical guidance and justification for future experiments. Our calculations suggest that XPS experiments, if the required control can be achieved, have the exciting potential of providing resolution for differentiating protonated water clusters with solvation shells of up to 14 water molecules.

## 3 Conclusion

In summary, we systematically studied the 1*s* CEBEs of B, C, N, and O elements with core-hole PPs from derivation and implementation within the real-space KS-DFT scheme. The results showed that real-space KS-DFT using PPs under Dirichlet boundary conditions can provide accurate binding energies as the localized-orbital-based, all-electron calculations. Furthermore, we proposed an additional refinement step for total energies using the B3LYP hybrid functional that generally improves the accuracy of the predicted binding energy shifts. In addition, the state-of-the-art real-space PP calculation method^62,80−82^ exhibits high computational efficiency, making it possible to predict binding energies for large-scale systems (>10,000 atoms) in the future.

## Supporting Information Available

Calculated Mulliken charges, binding energy shifts, and schematic illustrations of selected O in protonated water and binding energy shifts of N refined by different coefficients of Hartree–Fock exchange in B3LYP (PDF)
DSCF_DATA.tar: structures of clusters and input files of FHI98PP (ZIP)
