# Supplementary material for: Real-Space Pseudopotential Method for the Calculation of 1s Core-Level Binding Energies
Source: J Chem Theory Comput. 2022 Aug 29;18(9):5471–8. doi: 10.1021/acs.jctc.2c00474 (PMC9476661; doi:10.1021/acs.jctc.2c00474)
Supplement: Supplementary file 1 — ct2c00474_si_001.pdf [file ct2c00474_si_001.pdf]

Supporting Information

“Real-Space Pseudopotential Method for the Calculation of 1*s* Core-Level Binding Energies”

Qiang Xu[1], David Prendergast[2], and Jin Qian[1,*]

[1] *Chemical Science Division, Lawrence Berkeley National Laboratory, Berkeley, California 94720, USA.*

[2] *Molecular Foundry, Lawrence Berkeley National Laboratory, Berkeley, California 94720, USA.*

Email: jqian2@lbl.gov

Contents

This file includes:

1. Detailed results of relative 1*s* binding energies of molecules for B, C, N, and O elements (Table S1).
2. Discussions of energy refinement step by using different fractions in B3LYP (Figure S1 and Table S2).
3. Structural illustrations of protonated water clusters (Figure S2).

1. Detailed results of relative 1*s* binding energies of molecules for B, C, N, and O elements.

To further quantify the calculation the accuracy of binding energies, we provide the specific structural information, Mulliken charges, binding energies and their shifts, the mean absolute error (MAE) and mean error (ME) in Table S1. Note that Figure 3 and Figure 4 in the main text are plotted using the following raw data.

Table S1: Mulliken charges on B, C, N, and, O are calculated by AE-PBE. Relative binding energy shifts (eV) for 1s core hole (CH) of B, C, N, and O elements performed by AE-PBE, AE-B3LYP, PP-PBE, and PP-PBE (B3LYP) in comparison with the experiments. Underlines highlight the results that are closest to the experimental values. The absolute binding energies are listed in parentheses.

1s CH	Cluster	Charge	Exp.[2]	AE-PBE	AE-B3LYP	PP-PBE	PP-PBE(B3LYP)
B	$P(CH_3)_3BH_3$	-0.44	0.00 (192.93)	0.00 (192.26)	0.00 (193.24)	0.00 (191.60)	0.00 (191.77)
	$H_3BN(CH_2CH3)_3$	-0.39	0.27 (193.2)	0.20	0.26	0.24	0.29
	BH_3CO	-0.41	2.17 (195.10)	2.14	2.18	2.17	2.21
	$B(CH_2CH_3)_3$	0.15	2.57 (195.5)	2.15	2.29	2.18	2.35
	$B(OC_2H_5)_3$	0.13	4.67 (197.6)	4.28	4.70	4.42	4.87
	$B(OCH_3)_3$	0.13	4.87 (197.8)	4.53	4.96	4.68	5.13
	BBr_3	0.02	6.07 (199.0)	-	-	5.83	6.17
	BCl_3	0.11	6.87 (199.8)	-	-	6.45	6.88
	BF_3	0.44	9.87 (202.8)	9.22	9.84	9.35	10.01
	MAE (B)			0.27	0.06	0.23	0.11
C	CH_3SiH_3	-0.37	0.00 (290.31)	0.00 (289.78)	0.00 (290.68)	0.00 (288.16)	0.00 (288.15)
	CH_4	-0.43	0.49 (290.8)	0.39	0.47	0.43	0.53
	CH_3SH	-0.28	1.10 (291.41)	1.09	1.12	1.09	1.14
	CH_3OH	-0.09	2.11 (292.42)	1.88	2.02	2.04	2.18
	H_2NCN	-0.07	3.15 (293.46)	2.83	3.13	2.98	3.30
	HCN	-0.10	3.19 (293.5)	3.04	3.23	3.13	3.32
	NH_2CHO	0.14	4.14 (294.45)	3.60	4.02	3.89	4.30
	$HNCO$	0.21	5.58 (295.89)	5.07	5.67	5.34	5.96
	CO	-0.05	5.89 (296.2)	5.87	6.27	6.01	6.36
	CH_2F_2	0.29	6.05 (296.36)	5.48	5.84	5.78	6.12
	CCl_4	0.01	6.08 (296.39)	6.00	6.08	5.89	6.05
	CO_2	0.31	7.38 (297.69)	6.78	7.50	7.10	7.82
	CHF_3	0.41	8.79 (299.1)	8.04	8.58	8.44	8.96
	COF_2	0.38	9.33 (299.64)	8.48	9.20	8.89	9.62
	CF_4	0.44	11.54 (301.85)	10.53	11.26	11.00	11.72
	MAE (C)			0.38	0.12	0.20	0.18
N	$(C_2H_5)_2NH$	-0.18	0.00 (404.58)	0.00 (403.84)	0.00 (404.62)	0.00 (401.86)	0.00 (401.64)
	$H_2N(CH_2)_3OCH_3$	-0.13	0.30 (404.88)	0.35	0.29	0.35	0.28
	$CH_3CH_2NH_2$	-0.29	0.35 (404.93)	0.39	0.45	0.39	0.46
	$(CH_3)_3CCN$	-0.05	0.48 (405.06)	-	-	0.41	0.65
	CH_3NH_2	-0.30	0.57 (405.15)	0.62	0.68	0.66	0.73
	$H_2N(CH_2)_3OH$	-0.29	0.70 (405.28)	0.46	0.55	0.52	0.61
	NH_3	-0.04	1.02 (405.60)	1.00	1.13	1.08	1.22
	CH_3CN	-0.44	1.02 (405.60)	0.83	1.02	0.89	1.09
	CH_3SCN	-0.02	1.42 (406.0)	-	1.15	1.14	1.22
	$ClCH_2CN$	-0.02	1.49 (406.07)	1.25	1.46	1.31	1.53
	NH_2CHO	-0.16	1.72 (406.30)	1.91	2.01	2.00	2.11
	Cl_3CCN	-0.23	1.86 (406.44)	1.87	2.01	1.95	2.10
	$HNCO$	0.00	1.86 (406.44)	1.47	-	1.49	1.82
	$ClCN$	-0.02	1.87 (406.45)	1.50	1.72	1.52	1.76
	HCN	-0.04	2.22 (406.8)	2.24	2.39	2.28	2.44

	MAE (N)			0.14	0.12	0.15	0.14
O	$S(CH_3)_2O$	-0.50	0.00 (536.67)	0.00 (535.71)	0.00 (536.41)	0.00 (534.46)	0.00 (534.09)
	$(H_2N)_2CO$	-0.32	0.52 (537.19)	0.42	0.59	0.51	0.65
	NH_2CHO	-0.29	1.07 (537.74)	0.95	1.05	0.99	1.08
	CH_3CHO	-0.22	1.95 (538.62)	1.84	1.95	1.85	1.95
	$(CH_3)_2O$	-0.31	2.36 (539.03)	2.12	2.23	2.15	2.25
	CH_3OH	-0.09	2.53 (539.2)	2.46	2.57	2.49	2.58
	CH_2O	-0.21	2.77 (539.44)	2.86	2.91	2.87	2.90
	$COCl_2$	-0.12	3.05 (539.72)	-	3.14	2.95	3.14
	H_2O	-0.42	3.25 (539.92)	3.14	3.35	3.26	3.46
	HNCO	-0.17	3.49 (540.16)	3.55	3.72	3.60	3.77
	H_2CCO	-0.12	3.58 (540.25)	3.49	3.61	3.56	3.68
	OCS	-0.08	3.63 (540.30)	-	3.86	3.73	3.91
	COF_2	-0.19	4.10 (540.77)	3.86	4.16	3.90	4.17
	BH_3CO	-0.01	5.38 (542.05)	-	-	5.00	5.35
	CO	-0.05	5.90 (542.57)	6.00	6.12	6.04	6.14
	MAE (O)			0.11	0.10	0.11	0.12
Total	MAE			0.23	0.10	0.16	0.14
	ME			-0.20	0.02	-0.12	0.11

2. **Discussions of energy refinement step by using different fractions in B3LYP**

To assess the accuracy of binding energy shifts calculated by different coefficients of the exact exchange, Hartree-Fock (HF) exchange, in the hybrid functionals for energy refined step, we introduced the scaling parameter λ for the exchange part of B3LYP functional:

$$E_{XC}(\lambda) = 0.2\lambda E_X^{HF} + \frac{1-0.2\lambda}{0.8}\left(0.08E_X^{Slater} + 0.72E_X^{B88}\right) + 0.19E_C^{VWN1RPA} + 0.81E_{XC}^{LYP}$$

where $\lambda \in [0,5]$, and $\lambda = 0$ for density functional (no HF), $\lambda = 1$ for B3LYP and $\lambda = 5$ for full HF with correlation functionals. Plots of the binding energy shifts for N are calculated by different parameters and presented in Figure S1. We find that the density functional ($\lambda = 0$, black square) and B3LYP ($\lambda = 1$, red circles) results agree better with the experimental data than those obtained using other parameters. This is most likely the error cancellation between exchange and correlation[1], as the full HF ($\lambda = 5$, pink diamond) generally gives the worst result. As listed in Table S2, B3LYP ($\lambda = 1$) and the density functional ($\lambda = 0$) show the lowest MAE and ME, respectively, for the molecules' binding energy shifts of N.

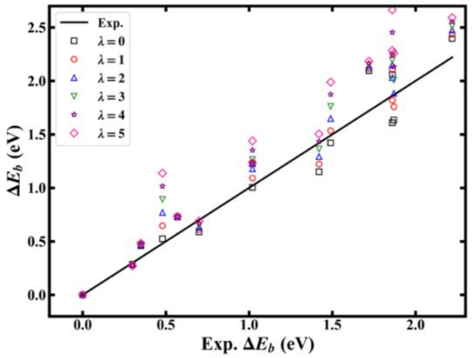

Figure S1: the refined binding energy shifts by different exchange-correlation functionals.

Table S2: The MAEs and MEs of the molecules' binding energy shifts (eV) of N by different parameters (λ) in the refined exchange-correlation functionals.

λ	0	1	2	3	4	5
MAE (eV)	0.15	0.14	0.16	0.21	0.26	0.31
ME (eV)	0.02	0.08	0.13	0.19	0.25	0.31

3. **Structural illustrations of protonated water clusters**

In Figure S2, we provide the optimized structures obtained in Refs.[3,4] for the calculation of O 1s binding energy shift in protonated water clusters $[H_3O^+ ...(H2O)_n, n \leq 20]$.

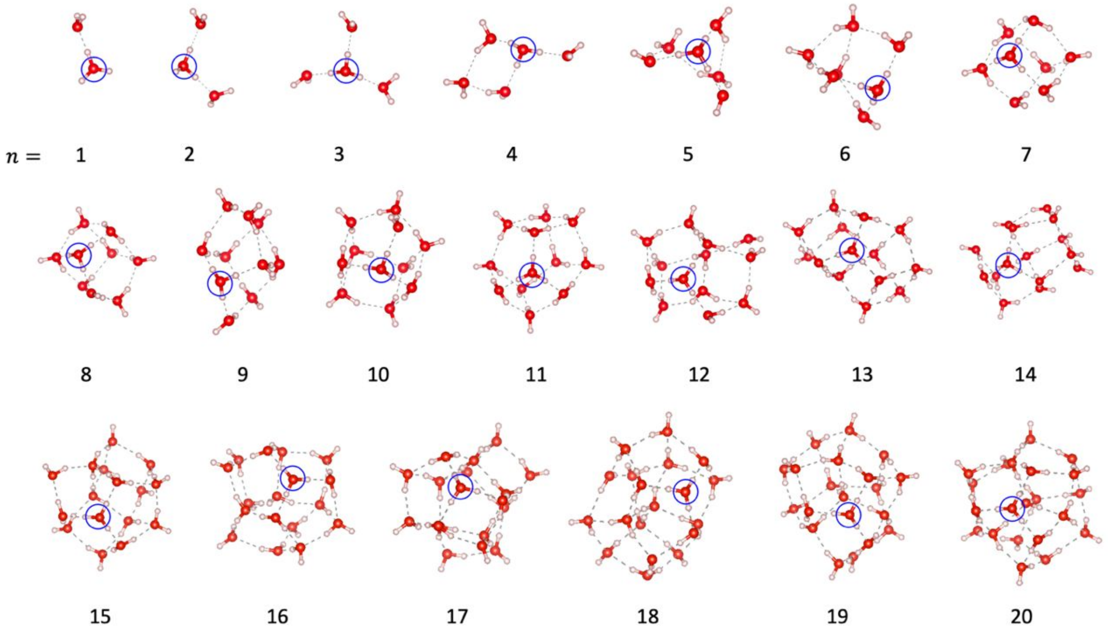

Figure S2: the structures of protonated water clusters $[H_3O^+ ...(H2O)_n, n \leq 20]$, where the blue circle denotes the O element with 1s core hole.

Reference

1. Furche, F. Molecular tests of the random phase approximation to the exchange-correlation energy functional. *Phys. Rev. B* **64**, 195120 (2001).
2. Jolly, W. L., Bomben, K. D. & Eyermann, C. J. Core-electron binding energies for gaseous atoms and molecules. *At. Data Nucl. Data Tables* **31**, 433–493 (1984).
3. Hodges, M. P. & Wales, D. J. Global minima of protonated water clusters. *Chem. Phys. Lett.* **324**, 279–288 (2000).
4. The Cambridge Energy Landscape Database. https://www-wales.ch.cam.ac.uk/CCD.html.
